# Effects of Two Polysaccharides From Traditional Chinese Medicines on Rat Immune Function

**DOI:** 10.3389/fvets.2021.703956

**Published:** 2021-11-12

**Authors:** Yu Lei Wang, Xiang Hua Shu, Xue Zhang, Yong Bo Liu, Ya Jing Zhang, Tao Lv, Xin Huang, Chun Lian Song

**Affiliations:** ^1^College of Veterinary Medicine, Yunnan Agricultural University, Kunming, China; ^2^College of Veterinary Medicine, South China Agricultural University, Guangzhou, China

**Keywords:** angelica, radix isatidis, polysaccharides, immune function, rats

## Abstract

**Abstract:** The aim is to study the immune function effect of two polysaccharides extracted from traditional Chinese herbs on rats. Ultrasonic-assisted extraction was used to extract the polysaccharide from traditional Chinese medicines. MTT assay was used to determine the effects of two polysaccharides on the conversion of pig peripheral T lymphocytes. For this, 24 Sprague–Dawley rats were selected for the clinical trial and divided into groups B (blank), CK (cyclophosphamide inhibitory control), AP (angelica polysaccharide), and RIP (radix isatidis polysaccharide). Except for group B, other groups can induce the immunodeficiency by using cyclophosphamide. Rats of the AP and RIP groups were given gavage of 1 mL of AP and RIP. The blood was sampled from the eyeball on days 0, 7, 14, 21, 28, and 35, respectively, to determine immune cells, IgG and IgM of immunoglobulin, body weight, and spleen index.

**Results:** The average content of AP and RIP was 51.27 and 14.8%, and the extraction rate was 75.23 and 60.94%. The maximum stimulation index was 1.407 when the concentration of AP was 8,000 μg mL^−1^ and 1.5 when the concentration of RIP was 125 μg mL^−1^. Both kinds of polysaccharides can alleviate the decline of white blood cells, lymphocytes, monocytes, neutrophils, and serum IgG and IgM caused by cyclophosphamide. The two polysaccharides can regulate the rapid recovery of weight in immunosuppressed rats and increase the spleen index of immunosuppressed SD rats. The polysaccharides from the two traditional Chinese medicines can alleviate the immunosuppression caused by cyclophosphamide and promote the immune function of the body, which can be used as raw material resources of new veterinary medicine.

## Introduction

Angelica, first contained in *Agriculture God's Canon of Materia Medica (Shennong Bencao Jing)*, is a common Chinese herbal medicine of the dried roots of Angelica (the umbelliferae plants) (1). Its main active components include ferulic acid, ligustilide and their isomers, and polysaccharides (2–4). Modern pharmacological studies show that AP can improve immunity and show antitumor, antiradiation, antioxidation, and antiaging effects (5–7). Radix isatidis, the dried root of the isatis tinctoria (Cruciferous plant), recorded in *Agriculture God's Canon of Materia Medica*, has the effect of clearing heat and detoxifying, cooling blood, and tonifying the pharynx (8). Modern pharmacology indicates that radix isatidis has antiviral, anti-inflammatory, and antipyretic effects (9–11), and radix isatidis polysaccharide (RIP) is its major component (12). Polysaccharide from traditional Chinese medicine can improve the immunity of animals (13), and it is also reported to enhance the phagocytosis of the mono-macrophage system, activate the reticuloendothelium system, induce the proliferation of lymphocytes, recognize antigens, and improve the lethality of NK cells (14–16). Antitumor and chemical protective effects of ginseng polysaccharide have received a lot of attention. In a tumor-bearing mouse model, a sublethal dose of cyclophosphamide after treatment with 100 mg/kg Ganshan injection significantly reduced mortality and promoted recovery (17).

However, with the vigorous development and continuous innovation of animal husbandry, the extensive use of antibiotics in animal husbandry production has brought a huge threat to food and environmental safety. The issue of drug-resistant bacteria has been raised many times by the World Health Organization. In the future, antibiotics as animal feed additives will be controlled more strictly, which will become the biggest obstacle to the future development of animal husbandry (18). Therefore, two kinds of polysaccharides were extracted from traditional Chinese medicine in this experiment and used to explore their effect on the immune function of cyclophosphamide immunosuppressed rats so as to provide relevant experimental data for the development of veterinary drugs.

## Materials and Methods

### Ethics Statement

All animal protocols were approved and supervised by the Experimental Animal Ethics Committee of YunNan Agricultural University NO. YNAU2019llwyh012.

### Experimental Animals and Medicinal Materials

A total of 24 Sprague–Dawley (SD) rats, weighting 150 ± 10 g, equal in gender, were purchased from the SPF Experimental Animal Center of Kunming Medical University. Landrace, weighing 20–30 kg, were purchased from the Experimental Pig Farm of Yunnan Agricultural University.

Angelica and radix isatidis were purchased from the traditional Chinese medicinal material market in Yunnan Province and were identified and qualified by the associate professor Bai Weibing of Yunnan Agricultural University. Angelica originated from Qujing, Yunnan, and radix isatidis originated from Luxi County, Honghe Prefecture, Yunnan.

This experiment has been approved by the Animal Ethics Committee of Yunnan Agricultural University.

### Test Reagents and Instruments

Test reagents included IL-2 kit (Lot No. 9670032121, ABclonal), IgG kit (Lot No. 9670012721, ABclonal), MTT (Lot No. 3580MG250, Biofrox), Erythrocyte lysate (Lot No. 67106955, Biosharp), fetal bovine serum (Lot No. 1828728, Gibco), and pig peripheral lymphocyte separation medium KIT (Lot No. LTS1110, Tianjin Hao Yang Biological Products Technology Co., LTD.)

Instruments included ultrasonic cleaning agent (JP-010D), Skymen cleaning equipment (Shenzhen Co., Ltd.), ultraviolet spectrophotometer (MODEL: EU-2600R, Shanghai Onlab Instrument Co., Ltd.), veterinary blood cell analyzer (MODEL BC-2800VET), carbon dioxide incubator (MODEL MCO-15AC, Sanyo Electric Co., Ltd).

### Experimental Methods

#### Extraction and Content Determination of Polysaccharide From Angelica and Radix Isatidis

The dried radix angelica medicinal material was crushed by a pulverizer and then passed through a 40-mesh screen to obtain powdered medicine. A total of 10 g powder was taken and added to 400 mL deionized water. Then, the mix was given ultrasonic extraction at 60°C for 15 min and then placed in a water bath at 80°C for 1 h. The mix was concentrated to 50 mL by rotary evaporation and added to 267 ml of 95% ethanol to make the alcohol content 80% and remained in a refrigerator overnight at 4°C. The solution was centrifuged at 2,000r min^−1^. The supernatant was taken, dried at 45°C, and stored at 4°C. The extraction method of RIP was the same as above. The content of polysaccharides was determined by phenol sulfuric acid assay. Briefly, accurately weigh 10 mg of glucose standard into a 250-mL volumetric flask, add double distilled water to the mark, and draw 0.2, 0.3, 0.4, 0.5, 0.6, 0.7, 0.8, and 0.9 mL, respectively, with distilled water to make up to 1.0 mL, add 1 mL of 5% phenol and 5.0 mL of concentrated sulfuric acid, shake well and cool down, and then measure the optical density at 490 nm by ultraviolet spectrophotometer after standing at room temperature for 20 min. The 1.0 ml of water performed as the blank control group. The standard curve was made with polysaccharide weight as abscissa and optical density value as ordinate. The regression equation was Y = 14.031X-0.002 (*R*^2^ = 99.75%), indicating a good linear relationship and accessible standard curve.

#### Transformation Experiment of T Lymphocyte

Landrace were adapted to the environment for 3 d and were taken for blood samples via the anterior vena cava. The peripheral blood lymphocytes of pigs were separated according to the instructions of the Tianjin Haoyang Lymphocyte Separation Kit (http://www.tbdscience.com/ProductsDetail.aspx?para=60-75A317BF-BEB1-4943-A8FF-4FF07F0B7D0D). Trypan blue staining was used for cell count (observation under microscopy of 40X). Then, lymphocytes proliferation experiment was performed. In detail, lymphocytes were inoculated evenly into a 96-well cell plate; each well contains about 10^5^-10^6^ cells. A total of four experimental groups were set up (I, II, III, IV), and each experimental group was repeated four times.

Cell suspension and different concentrations of TCM polysaccharide (31.25–8,000 μg/mL) were added to group I. In group II, only cell suspension was added. RPMI1640 complete culture medium and different concentrations of TCM polysaccharide were added to group III. In group IV, only RPMI1640 complete medium was added. Continue to incubate for 44 h under 5% CO_2_, and then, add 5 μL of MTT to each well under dark conditions. After continuing to cultivate for 4 h, then measure the absorbance value at 570 nm by UV spectrophotometer. The experiment was repeated three times. Transformation experiment of T lymphocyte.

Stimulus Index (SI) were calculated as follows:


$$\begin{array}{l} SI=\left\{\left(OD_{\text{I}}-OD_{\text{III}}\right)/\left(OD_{\text{II}}-OD_{\text{IV}}\right)\right\}\times 100\% \end{array}$$

Experimental groups are shown in Table 1.

**Table 1:** Experimental grouping.

**Groups**	**Treatments**
Group I	Cell suspension, different concentrations of TCM polysaccharide
Group II	Cell suspension
Group III	RPMI1640 complete culture medium, different concentrations of TCM polysaccharide
Group IV	RPMI1640 complete culture medium

#### Rat Clinical Trails

Experimental groupings and treatment are shown in Table 2. On days 0, 7, 14, 21, 28, and 35, 2 mL of blood was collected from the eyeball vein of SD rats in each group to detect IgG and IgM. Anticoagulant tubes of 0.5 mL were used to collect 100 μL of blood from the eyeball vein to detect routine blood indicators. The SD rats were weighed before each blood collection and put to death after the last blood sampling, and their spleens were weighed and spleen index was calculated. Spleen index = (spleen weight/ rat weight) × 100%.

**Table 2:** Experimental animal grouping.

**Groups**	**Treatments**
Group B	The rats were given intraperitoneal injection of normal saline on days 4, 5, and 6 and gavage of drinking water on day 4.
Group CK	The rats were given intraperitoneal injection of CTX on days 4, 5, and 6 and gavage of drinking water on day 4.
Group AP	The rats were given intraperitoneal injection of CTX on days 4, 5, and 6 and gavage of angelica polysaccharide on day 4.
Group RIP	The rats were given intraperitoneal injection of CTX on days 4, 5, and 6 and gavage of RIP on day 4

*The concentration of cyclophosphamide (CTX) was 70 mg·kg^−1^·BW, dose of 1 mL, once per day. The dose concentrations of RIP and AP were 200 mg·kg^−1^·BW, dose of 1 mL, once per day*.

#### Statistical Analysis of Data

All data were recorded and preliminarily processed with an Excel database and then analyzed by SPSS19.0 analysis software. The data results are expressed as mean ± standard deviation. Analyze the difference of the parameters through the method of analysis of variance. The significant level of difference is *P* > 0.05 or *P* < 0.01.

## Results and Analysis

### Extraction Rate and Content Results of Polysaccharide From Traditional Chinese Medicine

Table 3 shows that AP content was 51.27 ± 0.21% with the extraction rate of 75.23%, and RIP was 14.80 ± 0.23% with the extraction rate of 60.94%.

**Table 3:** Extraction rate and content of traditional Chinese medicine polysaccharide.

**TCM**	**No**.	**Content (%)**	**Average content (%)**	**Extraction rate (%)**
Angelica	1	50.92	51.27 ± 0.21	75.23
	2	51.65		
	3	51.24		
Radix Isatidis	1	15.09	14.80 ± 0.23	60.94
	2	14.97		
	3	14.34		

### Experiment Results of T Lymphocyte Transformation

**Figure 1:**
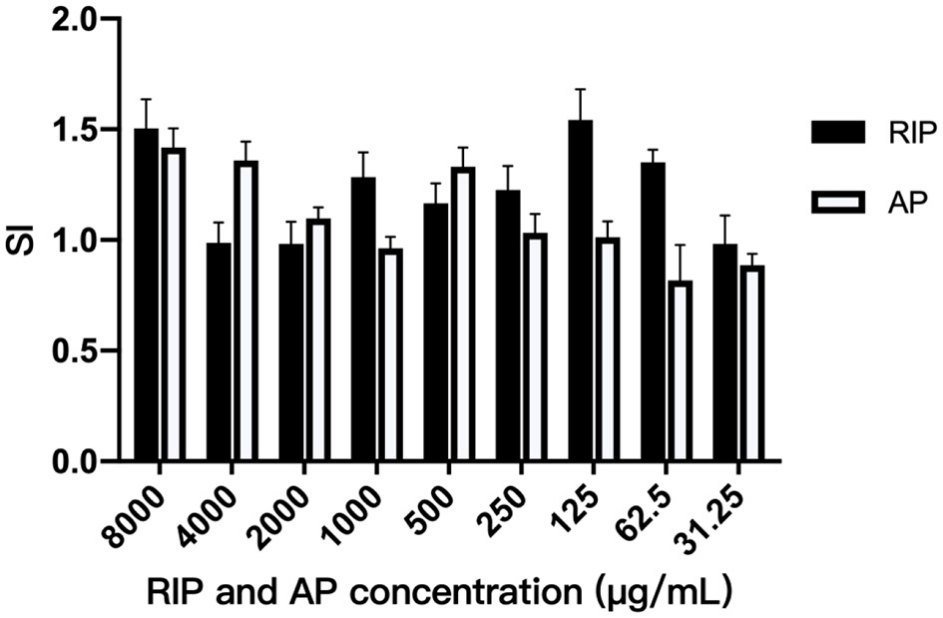
Experimental results of lymphocyte transformation from TCM polysaccharides.

It can be seen from Figure 1 that the SI were all greater than 1 when the concentration of AP was between 8,000 μg/mL and 250 μg/mL, indicating that the AP at the above concentration could improve the transformation rate of T lymphocyte. The highest SI was 1.42 at 8,000 μg/mL. SI was all greater than 1 when the concentration of RIP was between 8,000 μg/mL and 62.50 μg/mL, indicating that the T lymphocyte conversion could be improved by RIP at the above concentration. The SI was up to 1.5 at 125 μg/mL.

### Results of the Rat Clinical Trial

#### Effects of TCM Polysaccharides on Immune Cells

**Figure 2:**
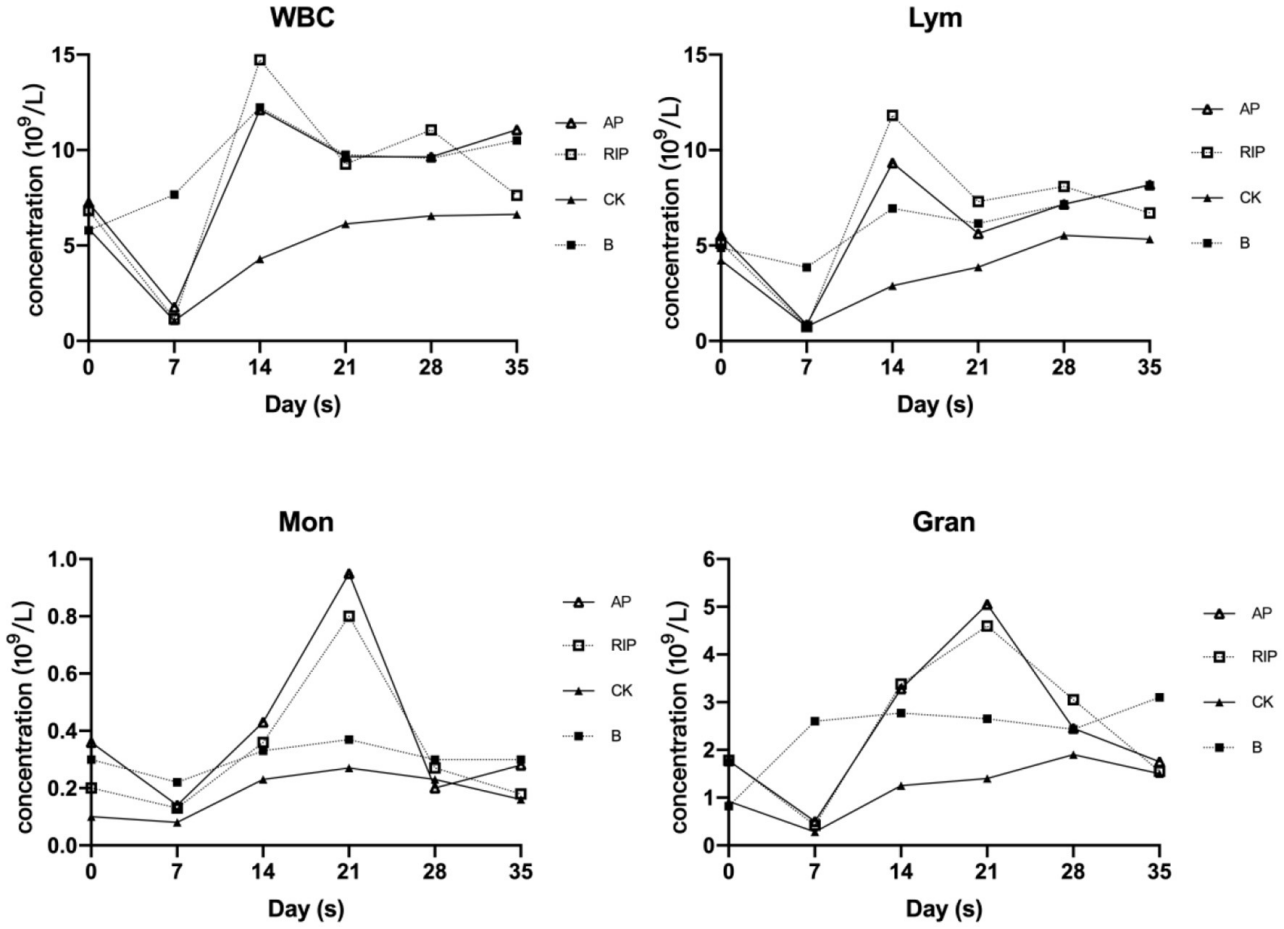
Changes of WBC, Lym, Mon, and Gran.

It can be seen in Figure 2, that white blood cells (WBCs), Lym (lymphocyte), Mon (monocyte), and Gran (granulocyte) of the group CK, and the TCM polysaccharide groups were all declining after cyclophosphamide injection, reaching the lowest value on day 7, and then began to rise. The group CK was lower than group B and TCM polysaccharide groups at each time point. The TCM polysaccharide groups played an obvious promoting role on the amount of WBC, Lym, Mon, and Gran.

#### Effects of TCM Polysaccharides on Immunoglobulin

**Figure 3:**
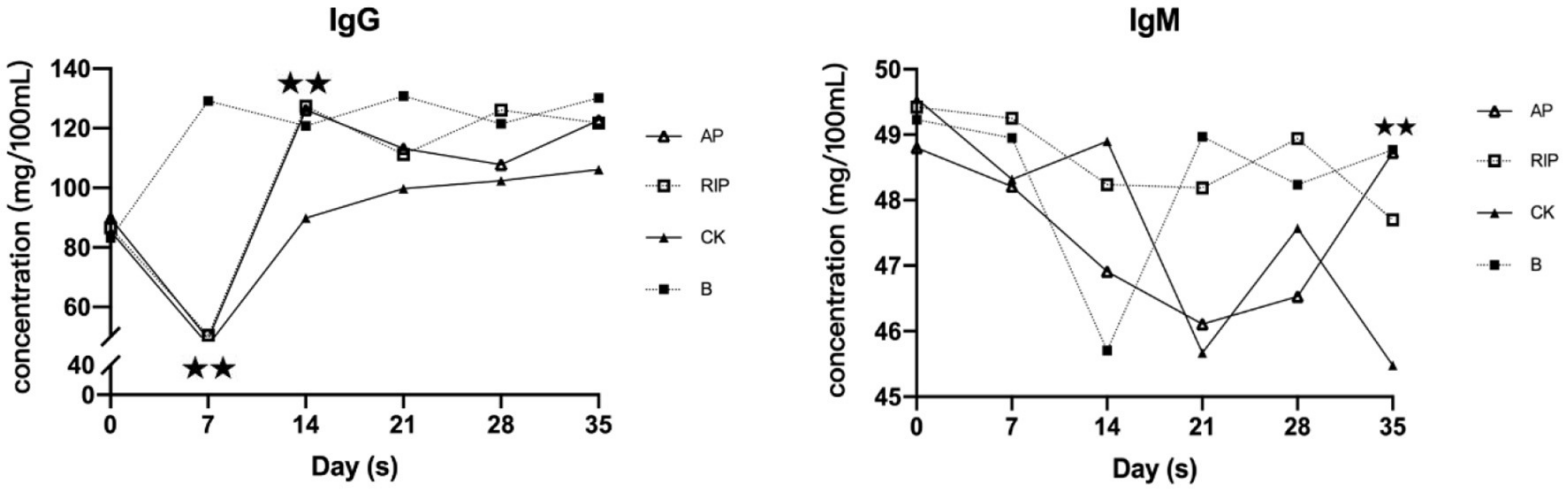
Changes of immunoglobulin G (IgG) and IgM. ⋆ showed *P* < 0.05, meaning there is significance; ⋆⋆ showed *P* < 0.01, meaning there is extreme significance.

As shown in Figure 3, IgG in group CK was decreased first and then increased. IgG of TCM groups and group CK were all significantly lower than that of group B (*P* < 0.01) on day 7. On the 14th day, IgG of the TCM groups and group B were significantly higher than that of group CK (*P* < 0.01), and IgG of the two TCM groups returned to the normal level after 28 days. On day 28, the IgM indexes of groups AP and RIP were significantly higher than that of group CK (*P* < 0.01).

#### Effects of TCM Polysaccharides on the Body Weight of SD Rats

**Figure 4:**
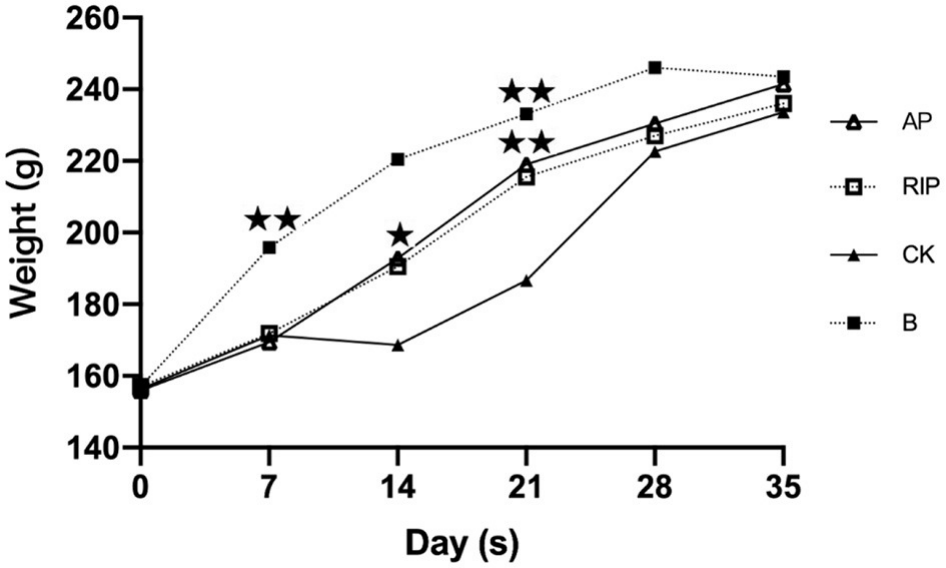
Comparison of the weight. ⋆ showed *P* < 0.05, meaning there is significance; ⋆⋆ showed *P* < 0.01, meaning there is extreme significance.

The body weight of SD rats in each experimental group showed an overall trend of increase as shown in Figure 4. There were significant differences between the TCM groups and group CK with group B in body weights on day 7 (*P* < 0.01). On day 14, there were significant differences between the groups AP, RIP, and CK (*P* < 0.05). On day 21, the body weight of group CK was significantly different from that of the other groups (*P* < 0.01). On day 35, there was no significant difference among groups in the weight of SD rats (*P* > 0.05), indicating that the weight of immunosuppressed rats had returned to the normal level.

#### Influence of TCM Polysaccharides on Spleen Index

**Figure 5:**
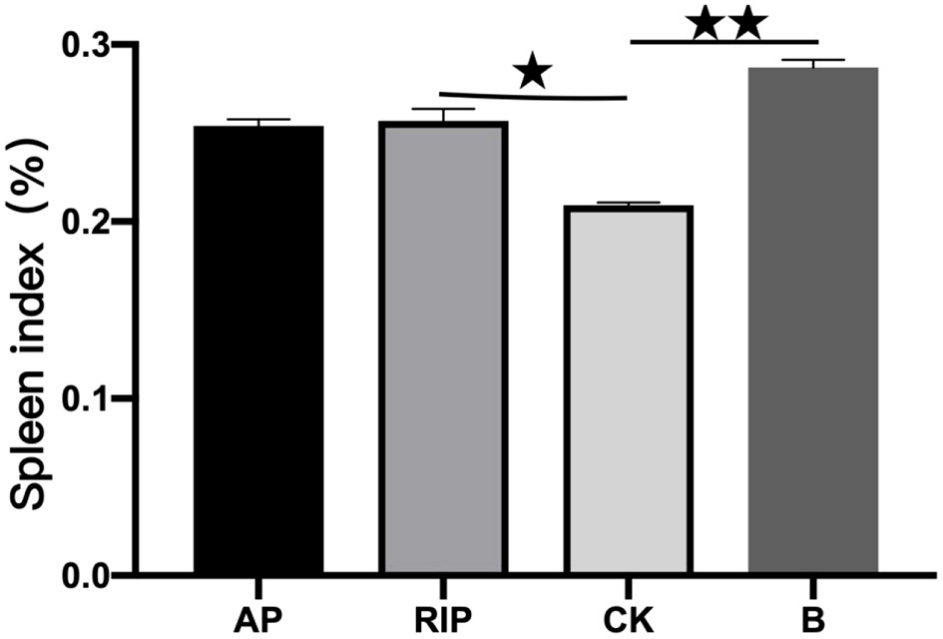
Comparison of the spleen index. ⋆ showed *P* < 0.05, meaning there is a significant difference compared with the RIP and CK groups; ⋆⋆ showed *P* < 0.01, meaning there is an extremely significant difference compared with the RIP and CK groups.

As shown in Figure 5, the spleen index of groups B, AP, and RIP were all higher than that of group CK. Compared with group CK, there were significant differences in group RIP (*P* < 0.05) and extremely significant differences in group B (*P* < 0.01). The results show that two kinds of TCM polysaccharides could increase the spleen index of immunosuppressed SD rats.

## Discussion

According to the report of Hou et al., AP, as the effective component of angelica, could significantly improve the red blood cell count and hemoglobin content of blood-deficient chickens. It could directly enrich the blood to increase red blood cells and hemoglobin, and it also regulates the hematopoietic factor, indirectly enriching the blood (19). According to Wang, the concentration of AP at 5 ~ 125 μg/mL can inhibit lymphatic proliferation induced by Concanavalin A. Serum transaminase (ALT and AST) levels were significantly reduced, and liver H&E staining showed that liver inflammation was reduced, indicating that ASP had potential liver-protection effects. ASP pretreatment could reduce liver injury caused by Concanavalin A in mice through anti-inflammatory and antioxidant effects (20). Du report the cell protection, antioxidant, and anti-inflammatory effects of RIP on mouse alveolar macrophages stimulated by lipopolysaccharide (LPS). Pretreatment with RIP could significantly prolong the survival time of mouse alveolar macrophages and inhibit the generation of reactive oxygen species (ROS) and lipid peroxidation of alveolar macrophages after LPS stimulation (11). Zhao finds that RIP can enhance non-specific immune function, humoral immunity, and cellular immunity in mice (21). These reports indicate that TCM polysaccharides have an enhanced effect on immunity that was similar to the results of this experiment.

Cyclophosphamide can degrade the activity of blood cell precursor in the bone marrow and reduce the number of red and white blood cells, thus leading to anemia, which is one of the main causes of low immunity (22). In this experiment, the number of red blood cells and hemoglobin in the two TCM groups were both increased to different degrees to enhance the immunity of immunosuppressed SD rats.

The body weight of the rats in the experimental group was on the rise, and the weight of group B was higher than those of the TCM groups and group CK. This indicates that cyclophosphamide affected the weight of SD rats, maybe due to the immunosuppression that affects appetite. Between days 14 and 21, the body weight of each drug group was higher than that of group CK, indicating that these two polysaccharides can eliminate the inhibiting effect of cyclophosphamide on the body weight of SD rats to a certain extent.

The results of the experiment show that RIP can increase the spleen index of immunosuppressed SD rats and improve their immunity. Spleen weight is closely related to the body's immunity (23), and spleen index refers to the ratio of spleen weight to body weight. Li et al. report that dendrobium polysaccharides can enhance the immune function of spleen immunity by increasing spleen index and phagocytosis of macrophages and stimulating lymphocyte proliferation in mice (24).

The maximum lymphocyte SI was 1.407 when the concentration of AP was 8,000 μg mL^−1^. SI was greater than 1.5 when the concentration of RIP was 8,000 μg mL^−1^ and 125 μg mL^−1^. The results show that the two polysaccharides increased the T lymphocyte conversion rate at the above conditions. At the same time, clinical experimental results also show that the two polysaccharides could significantly alleviate the decline of RBC, WBC, lymphocytes, neutrophils, and serum IgG caused by cyclophosphamide. The results show that the two polysaccharides could alleviate the immunosuppression caused by cyclophosphamide and promote the immune function of the body.

## Conclusions

Both AP and RIP can regulate the immune function of rats and have a value in new drug development and research.

## Data Availability Statement

The original contributions presented in the study are included in the article/supplementary material, further inquiries can be directed to the corresponding author/s.

## Ethics Statement

The animal study was reviewed and approved by Animal Ethics Committee of Yunnan Agricultural University.

## Author Contributions

YW, XS, and CS conceived and designed the experiments. YW, XZ, and YL performed the experiments. YW, YZ, TL, and XH analyzed the experiments. YL and YW contributed to the reagents and materials and wrote the paper. All authors read and approved the manuscript.

## Funding

This work was supported by Science and Technology Innovation Center Demonstration Plan (Kunming Science and Technology Innovation Center for Animal Epidemic Prevention and Control, KKJZ No. 2019-1-N-25318000003525). A major science and technology project in Yunnan Province Construction and Application of Technical System for Prevention and Control of Important Pig Diseases in Yunnan Province (202102AE090007).

## Conflict of Interest

The authors declare that the research was conducted in the absence of any commercial or financial relationships that could be construed as a potential conflict of interest.

## Publisher's Note

All claims expressed in this article are solely those of the authors and do not necessarily represent those of their affiliated organizations, or those of the publisher, the editors and the reviewers. Any product that may be evaluated in this article, or claim that may be made by its manufacturer, is not guaranteed or endorsed by the publisher.
